# Single-cell analysis reveals that GFAP^+^ dedifferentiated Schwann cells promote tumor progress in PNI-positive distal cholangiocarcinoma via lactate/HMGB1 axis

**DOI:** 10.1038/s41419-025-07543-x

**Published:** 2025-03-27

**Authors:** Ziyang Zu, Chong Zhang, Jianxiang Shi, Kunlun Chen, Hongwei Tang, Kaizhao Hu, Enchi Liu, Chengyang Ji, Ruo Feng, Xiaojing Shi, Wenlong Zhai

**Affiliations:** 1 Department of Hepatobiliary and Pancreatic Surgery, The First Affiliated Hospital of Zhengzhou University, Zhengzhou, 450052 China; 2 Precision Medicine Center, Henan Institute of Medical and Pharmaceutical Sciences & BGI College, Zhengzhou University, Zhengzhou, 450052 China; 3 Henan Engineering Technology Research Center of Organ Transplantation, Zhengzhou Engineering Laboratory of Organ Transplantation Technique and Application, The First Affiliated Hospital of Zhengzhou University, Zhengzhou, 450052 China; 4 Key Laboratory of Technology of Drug Preparation (Zhengzhou University), Ministry of Education of China, Key Laboratory of Henan Province for Drug Quality and Evaluation, Institute of Pharmaceutical Sciences, Zhengzhou University, Zhengzhou, 450001 China; 5 Tianjian Laboratory of Advanced Biomedical Sciences, Academy of Medical Sciences, Laboratory Animal Center, State Key Laboratory of Esophageal Cancer Prevention & Treatment, Zhengzhou University, Zhengzhou, 450052 China; 6 Department of Histology and Embryology, School of Basic Medical Sciences, Zhengzhou University, Zhengzhou, 450001 China

**Keywords:** Cancer microenvironment

## Abstract

Distal cholangiocarcinoma (dCCA) is a highly lethal malignancy that accounts for approximately 40% of patients with primary cholangiocarcinoma. Remarkable cellular heterogeneity and perineural invasion (PNI) are two typical features of dCCA. Deciphering the complex interplay between neoplastic and neural cells is crucial for understanding the mechanisms propelling PNI-positive dCCA progression. Herein, we conduct single-cell RNA sequencing on 24,715 cells from two pairs of PNI-positive dCCA tumors and adjacent tissues, identifying eight unique cell types. Malignant cells exhibit significant inter- and intra-tumor heterogeneity. We delineate the compositional and functional phenotypes of five Schwann cell (SC) subsets in PNI-positive dCCA. Moreover, our analyses reveal two potential cell subtypes critical to forming PNI: NEAT1^+^ malignant cells characterized by hypoxic propensity and GFAP^+^ dedifferentiated SCs featuring hypermetabolism. Further bioinformatics uncover extensive cellular interactions between these two subpopulations. Functional experiments confirm that lactate in the hypoxic tumor microenvironment can induce GFAP-dedifferentiation in SCs, which promotes cancer cell invasion and progression through upregulating HMGB1. Taken together, our findings offer a thorough characterization of the transcriptional profile in PNI-positive dCCA and unveil potential therapeutic targets for dCCA PNI.

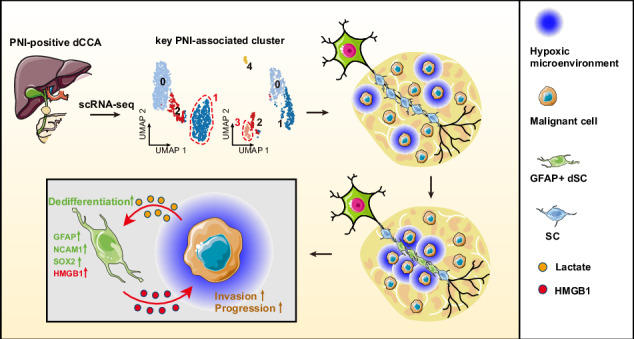

## Introduction

Cholangiocarcinoma (CCA) represents the most prevalent malignancy of the biliary system [1]. Depending on its anatomical location, CCA is typically categorized into intrahepatic CCA (iCCA), perihilar CCA (pCCA), and distal CCA (dCCA) [2, 3], with the latter localized to the common bile duct below the cystic duct insertion [2]. Over nearly three decades, the diagnostic morbidity and mortality of dCCA have continued to increase [4]. Due to early asymptomatic or nonspecific symptoms, many cases of dCCA are diagnosed in advanced stages, leading to limited available therapeutic options and an extremely poor prognosis. Surgical resection followed by adjuvant therapy may improve survival outcomes in patients with dCCA, but the high recurrence rate after dCCA surgery remains a challenge in clinical management [5]. Perineural invasion (PNI), refers to the characteristic biological process by which cancer cells invade nerves and spread along the perineurium [6]. In a recent cohort study, PNI was a common pathological phenomenon, present in 81.8% of dCCA cases [7]. Importantly, PNI has been recognized as a significant prognostic indicator affecting patients with resectable dCCA [8, 9].

Peripheral nerves partake in the constitution of a complicated tumor ecosystem comprising diverse cell populations, including Schwann cells (SCs). Physical contact between malignant cells and SCs has been found to promote directed movement and invasion of cancer cells [10]. Fuji-Nishimura et al. demonstrated that SCs facilitate colonization of pancreatic cancer in nerves by activating the epithelial-mesenchymal transition (EMT) pathway in tumor cells [11]. Recent research has illuminated that SCs could contribute to tumor progression by transitioning to a dedifferentiated state, analogous to their response to neurotrauma [10]. This reprogramming of SCs leads to the re-expression of glial fibrillary acidic protein (GFAP), neural cell adhesion molecule 1 (NCAM1), and L1 cell adhesion molecule (L1CAM), which can drive the development of PNI [10, 12, 13]. Presently, the initiator and tumor-promoting effect of dedifferentiated SCs (dSCs) in PNI-positive dCCA remains unclear. Consequently, a thorough comprehension of the cellular and molecular mechanism underlying neuromodulation of cancer progression is crucial for developing strategies for inhibiting tumor progression [6].

Herein, we employed the powerful technique of single-cell RNA sequencing (scRNA-seq) to profile PNI-positive dCCA and adjacent tissues, and identified two PNI-associated cellular components: NEAT1^+^ malignant cells and GFAP^+^ dSCs. We provided hitherto undocumented evidence that lactate in hypoxic tumor microenvironment (TME) could initiate GFAP-dedifferentiation of SCs, and the latter enhanced dCCA progression through upregulating high mobility group box 1 (HMGB1). Taken together, our findings offer an exhaustive transcriptomic overview and elucidate the intercellular interaction between malignant cells and SCs in PNI-positive dCCA, revealing potential therapeutic vulnerabilities in dCCA PNI.

## Results

### Single-cell transcriptomic profiling uncovered the spectrum of cell populations in human PNI-positive dCCAs

**Fig. 1:**
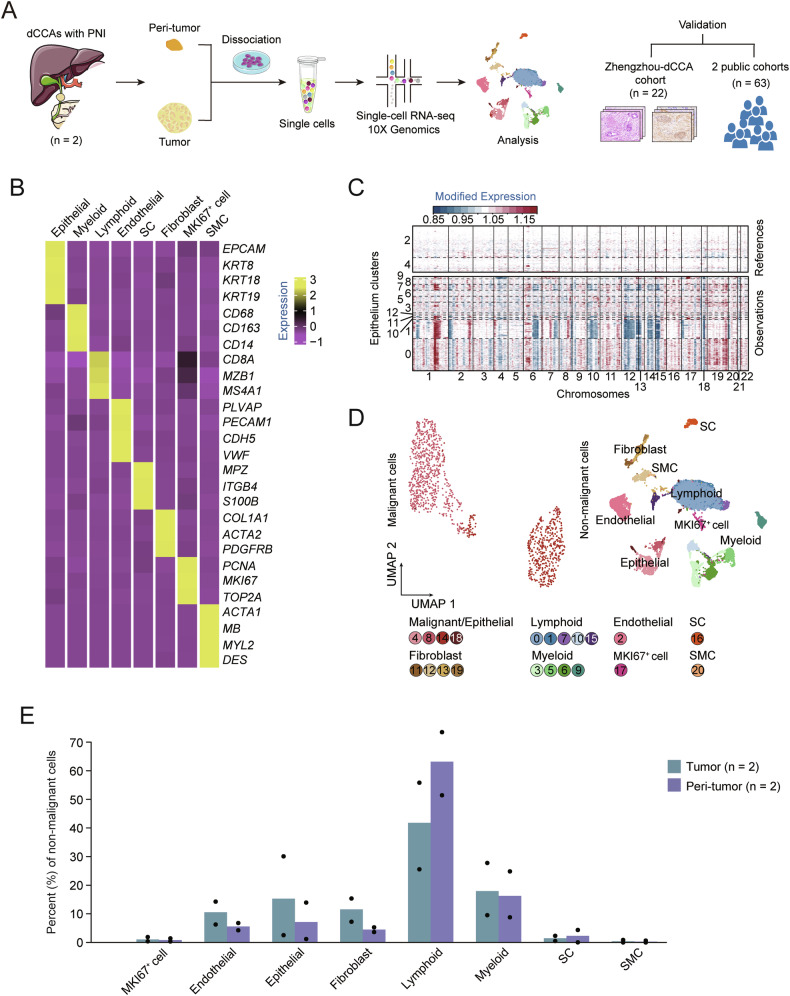
scRNA-seq profiling of 2 dCCAs. **A** Schematic representation of the experimental strategy. Part of the pictures were adapted from Servier Medical Art (http://smart.servier.com). **B** Heatmap showing the expression of marker genes in the indicated cell types. **C** Chromosomal landscape of inferred large-scale CNVs in normal epithelial cells (top) and potentially malignant cells (bottom) from 2 dCCA samples. Rows represent individual cells and columns represent chromosomal positions. Amplifications (red) or deletions (blue) were inferred by averaging expression over 100-gene stretches on the respective chromosomes. **D** Uniform manifold approximation and projection (UMAP) plot of malignant and non-malignant cells from 2 dCCA samples. **E** Boxplot showing the fraction of non-malignant cells in tumor and peri-tumor tissues.

To comprehensively understand the tumor ecosystem in dCCA with PNI, we conducted scRNA-seq on tumor and paired adjacent non-neoplastic tissues from two untreated PNI-positive dCCAs (Fig. 1A). Detailed clinicopathological features of the study population are listed in Table S1. Following quality control and filtering, single-cell transcriptome profiles were obtained for 24,715 cells. Eight primary cell types were determined informed by established marker genes, including epithelial cells (2696, 10.9%), myeloid cells (4212, 17.0%), lymphoid cells (13,161, 53.3%), endothelial cells (1946, 7.9%), SCs (472, 1.9%), fibroblast (1919, 45.6%), MKI67^+^ cells (227, 0.9%), and smooth muscle cells (SMCs, 82, 0.3%, Fig. 1B). Subsequently, we extracted all epithelial cells and identified 13 subclusters through reclustering analysis. Clusters 2 and 4 were considered normal epithelium and served as a normal reference for copy number variation (CNV) analysis due to their predominant distribution in adjacent noncancerous tissues (Fig. 1C, Fig. S1A). A total of 1203 malignant cells expressing high levels of KRT19 were inferred and further reclustered (Fig. S1B). Figure 1D illustrates the original 21 cell clusters for all cells. Consistent with previous dCCA studies [14, 15], non-malignant cells (excluding SMCs) exhibited inter- and intratumoral heterogeneity across different tissues. For instance, endothelial cells, epithelial cells, myeloid cells, and fibroblasts were heavily infiltrated in tumors, whereas lymphoid cells and SCs were predominantly found in adjacent biliary ductal tissues (Fig. 1E). Moreover, to validate our findings, we used CIBERSORTx [16] to deconvolute bulk RNA-seq data from a broader cohort of CCA and normal samples. The relative abundance of endothelial cells, fibroblasts, and SCs in our samples conformed with estimates from the TCGA-CHOL dataset. However, epithelial cells and immune cells displayed discrepant patterns (Fig. S1C).

### Highly heterogeneous hypoxic patterns of malignant cells and their contribution to the PNI-positive dCCA microenvironment

**Fig. 2:**
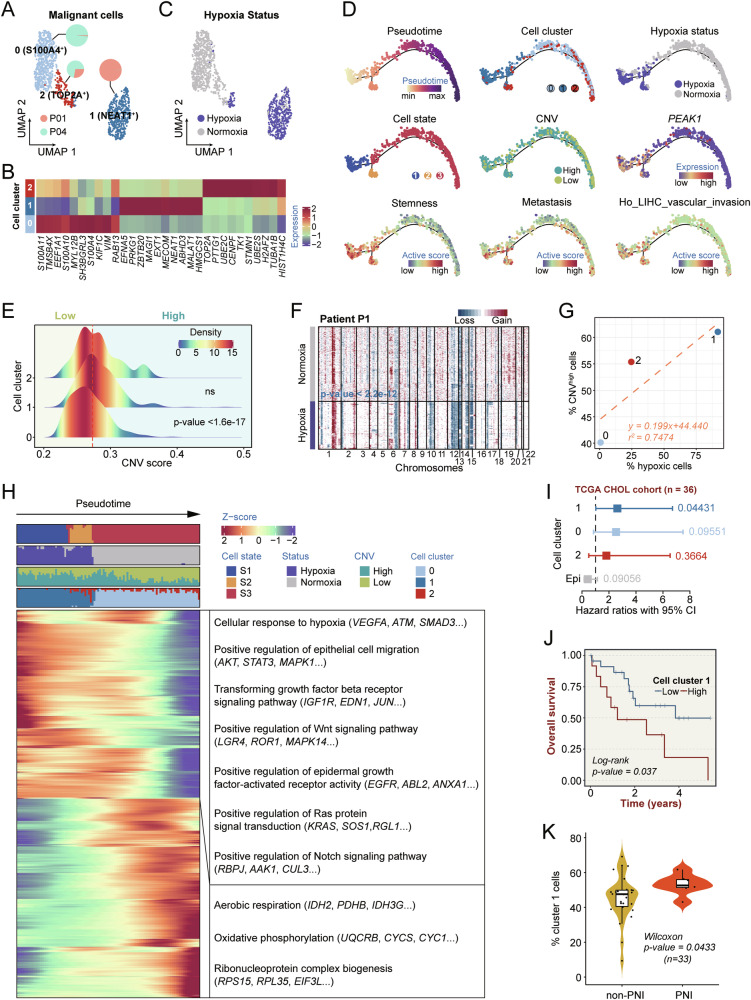
Transcriptional signatures and hypoxia heterogeneity of malignant cells. **A** UMAP plot of three malignant subtypes. Pie charts for each subtype showing the contributing percentage of cells from each patient. **B** Heat map showing the top differentially expressed genes (DEGs) in each malignant subtype. **C** UMAP plot of malignant cells colored by hypoxia status. **D** Semisupervised pseudotime trajectory of malignant subtypes inferred by Monocle2. Trajectory is colored by pseudotime (top left), cell subtypes (top middle), hypoxia status (top right), cell states (left), CNV levels (middle), the expression dynamics of a selected marker gene *PEAK1* (right), stemness signature scores (bottom left), metastasis signature scores (bottom middle), signature scores calculated based on the HO_LIVER_CANCER_VASCULAR_INVASION geneset (bottom right). **E** Malignant cells were grouped into different categories based on the CNV score. Ridgeline Plots show the distribution of CNV scores across different cell clusters. The red dashed line indicates the threshold value. **F** CNV inferred by scRNA-seq data in patient P1. **G** The percentage of hypoxic cells is positively correlated with the proportion of cells with high levels of CNVs. **H** Heatmap showing the scaled expression of DEGs across pseudotime trajectory in (**D**). Bar plots at the top of the heatmap are scale diagrams of different cell states, hypoxia status, CNV levels, and cell subtypes during pseudotime differentiation trajectory. **I** Association of relative cell abundance (estimated by CIBERSORTx) and patient survival using the TCGA-CHOL cohort (*n* = 36) by COX regression analysis. **J** Kaplan–Meier curves of TCGA-CHOL patients (*n* = 36) showing the survival rates grouped by the cell abundance in malignant cell cluster 1. The *P* value is calculated with two-sided log-rank test. **K** Violin plots displaying the cell abundance in malignant cell cluster 1 in non-PNI and PNI groups. non-PNI, *n* = 26 samples; PNI, *n* = 7 samples. The central mark indicates the median, and the bottom and top edges of the box indicate the first and third quartiles, respectively. The top and bottom whiskers extend the boxes to a maximum of 1.5 times the interquartile range. ns, not significant.

To characterize the tumor cell landscape in PNI-positive dCCA, malignant cells were subsequently clustered and divided into three primary subclusters (Fig. 2A, B). In alignment with previous findings in dCCA [14], malignant cells exhibited significant intra- and inter-tumor heterogeneity (Fig. 2A). The distinctive expression patterns within these three subpopulations are illustrated in Fig. 2B. Cluster 0 was enriched for cells that highly expressed genes in the S100 family, such as *S100A4*, *S100A10*, and *S100A11*. S100 protein family members have been commonly observed to be dysregulated in various tumors, including iCCA, and are critically implicated in carcinogenesis and cancer progression [17–19]. Cluster 1 was characterized by a prominent upregulation of *NEAT1* and *MALAT1*. These two adjacent long non-coding RNA genes have been extensively documented to be involved not only in activating multiple oncogenic mechanisms but also in conferring resistance to chemotherapeutics [20, 21]. *TOP2A* and *TK1*, both of which were substantially expressed in cluster 2, have been previously recognized as proliferative markers in many studies [22–24]. Hypoxia is a ubiquitous property of most solid cancers and is strongly linked to tumor metastasis and invasion [25]. We subsequently visualized the hypoxia statuses of malignant cells using the cellular hypoxia predicting framework (CHPF) [26]. Among these, most hypoxic cells were concentrated in cluster 1 (NEAT1^+^) malignant cells, with fewer found in cluster 0 (S100A4^+^) and cluster 2 (TOP2A^+^) (Fig. 2C). Additionally, to explore the influence of hypoxia on the evolutionary dynamics of malignant cells in PNI-positive dCCA, Monocle2 and CytoTRACE were employed to perform unsupervised cell trajectory analysis, both of which revealed a similar differentiation pathway of malignant cells originating from hypoxic cells (Fig. 2D, Fig. S2A), consistent with the conclusion drawn by Zhang et al. in glioblastoma [26]. Three cell states (S1–S3) were defined for pseudotime trajectory analysis based on Monocle2 (Fig. 2D). In terms of cellular status, hypoxic cells were primarily confined to S1 and S2 at the initial stage of differentiation, whereas normoxic cells were predominantly concentrated in S3. Regarding cell clusters, NEAT1^+^ malignant cells (cluster 1) dominated the S1 state, appearing at the earliest stage of pseudotime and exhibiting significantly higher stemness scores. We postulated that the high stemness of cluster 1 might be related to its deduced hypoxic state, according to previous studies [27–29]. Correspondingly, S100A4^+^ malignant cells (cluster 0) constituted the primary subcluster of the S3 state and were exclusively observed in the final stage of cell differentiation. Notably, TOP2A^+^ malignant cells (cluster 2) spanned across both S2 and S3 states, suggesting the presence of two distinct cell substrates within cluster 2 (Fig. 2D). Taken together, these findings indicated an orchestrated differentiation process of dCCA cells during PNI. PEAK1, a novel human pseudokinase, has recently been implicated in cancer pathogenesis [30]. We observed that PEAK1^+^ malignant cells were positioned at the beginning of the major branch and aligned well with cluster 1. Similarly, metastasis scores and hepatic vascular invasion scores were predominantly observed at the onset of differentiation. These findings suggested that cluster 1 might represent a key cell type with high invasiveness in PNI-positive dCCA.

To delve deeper into the relationship between hypoxia and the aggressive phenotype of PNI-positive dCCA, hypoxia-related signature genes from the CancerSEA database [31] and several hallmark gene sets, including EMT, IL2/STAT5, PI3K/AKT/mTOR, and KRAS signaling from MSigDB, were manually curated (Table S2). Given gene set variation analysis (GSVA) to determine the activity score of each malignant cell, the association between hypoxia and tumor invasion activities in PNI-positive dCCA was evaluated. The results showed that invasion score was significantly positively correlated with hypoxia (Fig. S2B). Utilizing the previously inferred single-cell CNV spectrum, we observed that clusters 1 and 2 exhibited higher CNV levels than cluster 0 (Fig. 2E). In addition, the extent of CNV accumulation was correlated with the hypoxic status of cells. As exemplified by malignant cells derived from patient P1, hypoxic malignant cells displayed significantly higher CNV levels than normoxic malignant cells, indicative of a more malignant trait. In this respect, high-frequency CNV events were enriched in certain chromosomes, such as chr6, chr12, and chr15 (Fig. 2F). We categorized all malignant cells into low and high groups based on CNV levels (Fig. 2D, E). The percentage of hypoxic cells within each malignant cell cluster demonstrated a positive correlation with the percentage of CNV^high^ cells in that cluster (Fig. 2G). In addition, pathway enrichment analysis using GSVA revealed that MTORC1 signaling, MYC targets, E2F targets, and EMT pathways were enriched in the CNV-high group (Fig. S2C). Overall, hypoxia and high CNV levels might be essential for preserving the malignant characteristics of cluster 1. To summarize the transcriptomic features of malignant cells, we integrated meta-information regarding cell cluster, hypoxic state, CNV status, and predicted trajectories. The cellular developmental process was divided into two distinct phases based on dynamic gene expression patterns (Fig. 2H). Correspondingly, the initial phase primarily comprised states S1 and S2, and there was a propensity for cluster 1 cells to transition to cluster 2 during this stage. This alteration was accompanied by downregulation of the hypoxia-induced gene *VEGFA* and the oncogenic driver, *AKT*, as well as diminished signaling pathways associated with hypoxia response and epithelial cell migration. In contrast, cluster 1 cells in the second phase exhibited a greater propensity to transform into cluster 0 and subsequently progress toward the S3 state, characterized by heightened expression of RPS15 and a shift in energy metabolism towards aerobic respiration (Fig. 2H).

To investigate the clinical implication of the malignant cell subtypes identified in our study, we estimated the proportion of epithelial cell subpopulations (including normal epithelial cells) within patient samples from the TCGA-CHOL cohort using CIBERSORTx (Table S3). Only the increased abundance of cluster 1 malignant cells showed a significant correlation with decreased overall survival (OS; Fig. 2I, J). We subsequently obtained similar results using GSE107943 as a validation dataset (Fig. S2D, Table S4). Furthermore, utilizing information on samples from the TCGA cohort containing patient PNI status, we discovered that cluster 1 malignant cells were significantly more abundant in CCA with PNI than CCA without PNI (Fig. 2K). These findings indicated that cluster 1 (NEAT1^+^) malignant cells, characterized by hypoxia propensity and higher levels of CNV, may be associated with PNI in dCCA.

### dSCs play a significant role in dCCA PNI

**Fig. 3:**
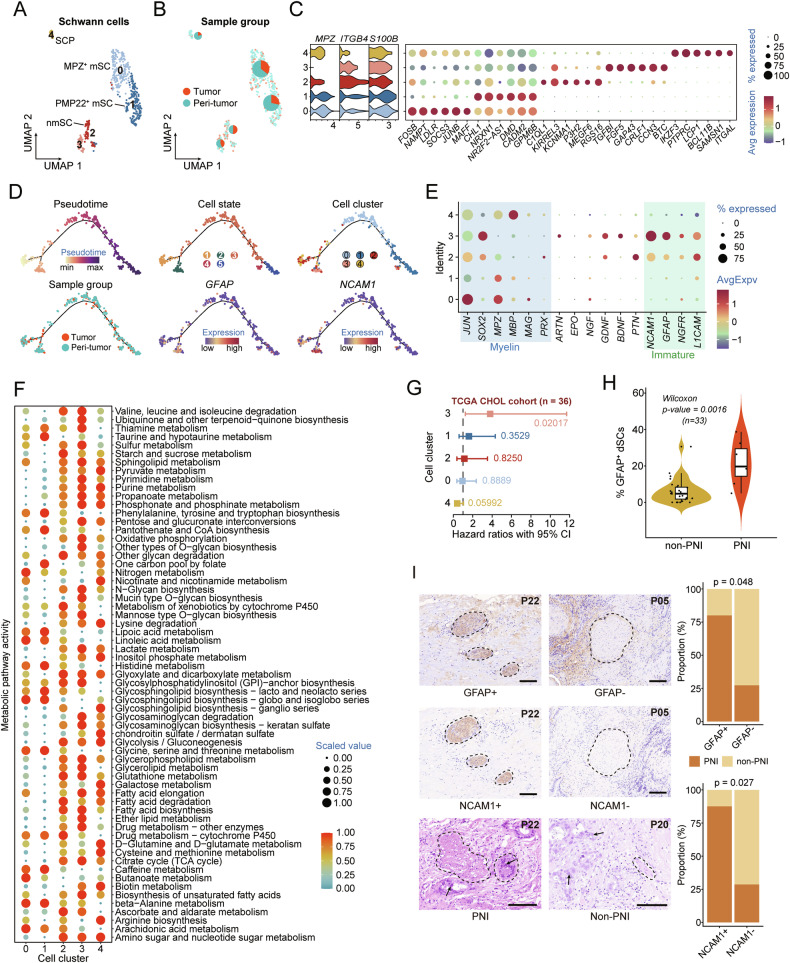
Transcription profiling of SCs in the TME of PNI-positive dCCA tissues. **A** UMAP showing the five subtypes of SCs, colored by subclusters. **B** Distribution of SCs in different sample groups on the UMAP. Pie chart showing the proportion of two sample groups in each SC subcluster. **C** Violin plots (left) displaying the representative expression pattern across different subtypes of SCs. Dot plot (right) showing the expression of the top six subtype-specific gene markers in each subtype. **D** Semisupervised pseudotime trajectory of SC subtypes by Monocle2. Trajectory is colored by pseudotime (top left), cell states (top middle), cell clusters (top right), sample groups (bottom left), and expression dynamics of two marker genes *GFAP* (bottom middle) and *NCAM1* (bottom right). **E** Dot plot illustrating the expression patterns of selected dSC gene markers in each SC subtype. **F** Dot plot showing the metabolic activity analysis of all SC subclusters by scMetabolism. The circle size and color darkness both represent the scaled metabolic score. **G** Association of relative cell abundance (estimated by CIBERSORTx) and patient survival using the TCGA-CHOL cohort (*n* = 36) by COX regression analysis. **H** Violin plots displaying the cell abundance in GFAP^+^ dSC in non-PNI and PNI groups. non-PNI, *n* = 26 samples; PNI, *n* = 7 samples. The central mark indicates the median, and the bottom and top edges of the box indicate the first and third quartiles, respectively. The top and bottom whiskers extend the boxes to a maximum of 1.5 times the interquartile range. **I** Representative images (top left) of immunohistochemistry (IHC) expression of GFAP and NCAM1 in patients from the Zhengzhou-dCCA cohort (*n* = 22). Representative images (bottom left) of H&E staining assays of PNI and non-PNI patients from the Zhengzhou-dCCA cohort. The experiment was repeated once with similar results. Nerves are highlighted with dotted lines and tumor cells with arrows. Scale bars, 100 μm. Bar plot (right) showing the positive proportion of IHC staining for GFAP and NCAM1 from PNI and non-PNI patients from the Zhengzhou-dCCA cohort.

SCs have been firmly established as a novel cell type within the TME, playing a specific and cancer-promoting role in PNI [32]. We focused our analysis on SCs in dCCA, performing unsupervised clustering on 472 cells, and identifying five distinct subclusters (Fig. 3A). Utilizing a marker gene list curated from the Tabula Sapiens portal [33] and previous literature by Kastriti et al. [34], we observed that clusters 0 and 1 exhibited overexpression of myelinating SC (mSC) markers like *EGR2*, *MPZ*, and *PMP22*. Cluster 2 displayed upregulation of well-defined non-myelinating SC (nmSC) markers such as *IGFBP5*, *TAGLN2*, *TPM1*, and *A2M*. Notably, cluster 4 preferentially expressed genes indicative of SC precursors (SCPs): *CD69*, *BTG1*, *CD52*, *CYBA*, and *LTB* (Fig. 3A, Fig. S3A). Among these clusters, MPZ^+^ mSCs (cluster 0), PMP22^+^ mSCs (cluster 1), and SCPs (cluster 4) were predominantly located in cancer-adjacent tissues. Conversely, nmSCs (cluster 2) and cluster 3 had a greater proportion of cells distributed within cancer tissues (Fig. 3B). Figure 3C illustrates the unique transcriptomic signatures of all SC subsets identified in dCCA.

To investigate the developmental pathways and potential roles of these distinct SC subclusters in dCCA with PNI, we first employed CytoTRACE to estimate the differentiation degree of each subcluster. As expected, the SCP cluster, representing multipotent embryonic progenitors for many neural cells [35, 36], possessed the highest differentiation score (Fig. S3B). Then, we reconstructed the SCs into a pseudotime trajectory using Monocle2, designating the SCPs as the starting point. Five distinct cell states (S1–S5) and a primary trajectory route were identified (Fig. 3D). We observed that cluster 3 was positioned in close proximity to the differentiation starting site and characteristically expressed the dedifferentiation markers *GFAP* and *NCAM1* (Fig. 3D). Furthermore, we found that cluster 3 also upregulated the myelin-related gene *SOX2* and the immature genes *NGFR* and *L1CAM* (Fig. 3E), aligning well with the reprogramming process of dSCs described by Jessen et al. [37]. Therefore, we classified cluster 3 as dSC. Previous studies have indicated that both mSCs and nmSCs can contribute to cancer progression by transitioning to the dSC phenotype characteristic of repair SCs in cancer [32, 37]. Our analysis further revealed that PMP22^+^ mSCs and certain nmSCs (specifically cluster S4) initially transitioned to cluster 0 (MPZ^+^ mSC) during the dedifferentiation process. Notably, this transition occurred with little up-regulation of immature genes, while *JUN* expression increased but *SOX2* remained relatively unchanged. Indeed, both *JUN* and *SOX2* are myelin suppressor genes. In contrast, *SOX2* expression became prominent during the dedifferentiation phase (Fig. 3D, E). These findings suggest that the abandonment of myelin differentiation in dSCs may precede the activation of the immature phenotype, and different negative regulators of myelination seem to act asynchronously. Overall, for PNI-positive dCCA, most SCs transition from SCPs to GFAP^+^ dSCs, traversing an intermediate state (Cluster 0, MPZ^+^ mSC). Ultimately, they may develop into PMP22^+^ mSCs or nmSCs. Our analysis provides a comprehensive ecological map and trajectory evolution of SCs in PNI-positive dCCA.

Gene ontology (GO) analysis revealed that MPZ^+^ mSCs were significantly enriched in neuron apoptosis processes, neuron death, and tumor necrosis factor-mediated signaling pathways, possibly reflecting the damage response induced by cancer cell invasion (Fig. S3C). Conversely, enriched GO terms for PMP22^+^ mSCs were associated with neural support and regeneration, including axonogenesis, axon development, and regulation of synapse maturation (Fig. S3C). nmSCs were characterized by a high level of extracellular matrix similar to fibroblasts, while GO terms of GFAP^+^ dSCs were enriched in cholesterol binding, lipid transfer activity, and phosphatidylcholine binding, indicating their higher metabolic properties (Fig. S3C). Finally, GO analysis of SCPs revealed their enrichment in pathways such as activation of the immune response, regulation of T cell activation, and neutrophil migration, suggesting a potential role in immune regulation (Fig. S3C). To further elucidate the metabolic landscape of SCs in PNI-positive dCCA, scMetabolism was employed [38] to systematically quantify metabolic activities at single-cell resolution. We computed metabolic pathway activity scores for all 63 metabolic pathways annotated in scMetabolism and found that GFAP^+^ dSCs exhibited higher metabolic activity (Fig. 3F, Fig. S3D). Among these pathways, pyruvate metabolism, lactate metabolism, glycerolipid metabolism, and fatty acid biosynthesis were markedly activated in GFAP^+^ dSCs (Fig. 3F).

To explore the influence of each SC cluster on dCCA prognosis, CIBERSORTx was applied to determine the percentage of diverse SC types across the TCGA-CHOL samples (Table S5). High infiltration of GFAP^+^ dSCs was associated with an inferior prognosis (Fig. 3G, Fig. S3E). Similar results were obtained in the GSE107943 validation cohort (Fig. S3F, Table S6). To study the contribution of GFAP^+^ dSCs to the occurrence of PNI in dCCA, the TCGA-CHOL samples were sorted into PNI and non-PNI groups founded on the presence or absence of concomitant PNI. We observed that the PNI group displayed significantly higher infiltration of GFAP^+^ dSCs (Fig. 3H). Additionally, 22 dCCA patients from the First Affiliated Hospital of Zhengzhou University were enrolled in our internal cohort (Zhengzhou-dCCA cohort). Hematoxylin and eosin (H&E) staining confirmed that all pathological sources were tumor tissues (Fig. S4A). Immunohistochemistry (IHC) analysis demonstrated that the positive rates of GFAP and NCAM1 proteins in the neural tissue of PNI samples were higher than those in non-PNI samples (Fig. 3I, Table S7). Collectively, these data suggest that GFAP^+^ dSCs possess high metabolic characteristics and play crucial roles in the PNI-positive microenvironment.

### Interactome landscape across NEAT1^+^ malignant cells and GFAP^+^ dSCs in the PNI-related dCCA microenvironment

**Fig. 4:**
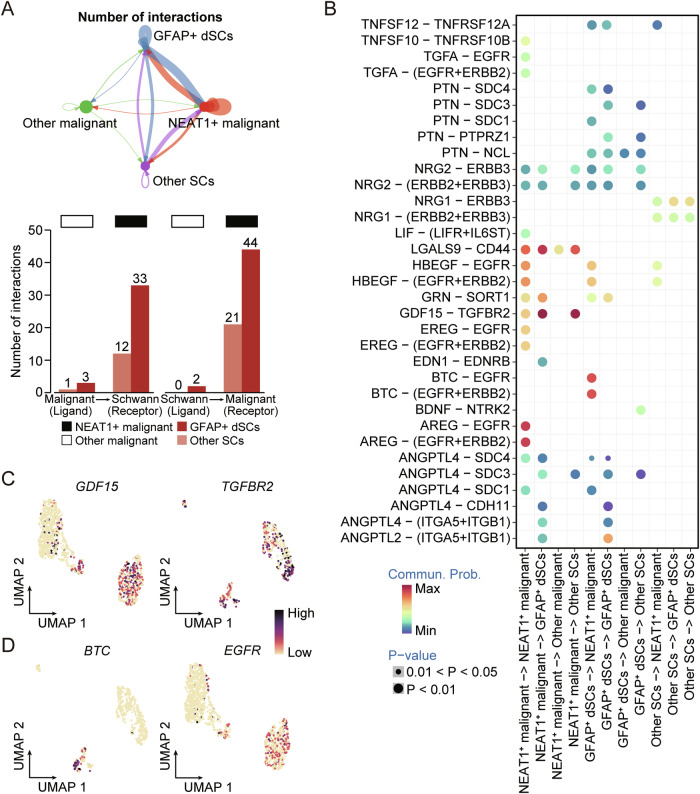
Cell-cell communication between malignant cells and SCs. **A** Cell-cell interaction network (top) of NEAT1^+^ malignant cells, other malignant cells, GFAP^+^ dSCs, and other SCs. The node size represents the number of interactions. The width of the edge represents the number of significant ligand–receptor interactions in two cell types. Bar plot (bottom) presenting the numbers of putative ligand-receptor pairs between malignant cells and SCs. **B** Bubble heatmap showing interaction strength for different ligand-receptor pairs. Dot size indicates the *P* value generated by the permutation test and dot color represents communication probabilities. Empty space indicates that the communication probability is zero. **C**, **D** UMAP plot showing expression levels of GDF15-TGFBR2 (**C**) and BTC-EGFR (**D**) ligand–receptor pairs in specific cell types.

To elucidate the crosstalk between NEAT1^+^ malignant cells and GFAP^+^ dSCs within the TME during PNI progression, we investigated intercellular communication by simulating ligand-receptor interactions using CellChat. A total of 116 pairs of interactions were identified across the four cell types we classified. Notably, NEAT1^+^ malignant cells and GFAP^+^ dSCs exhibited the highest number of interactions (Fig. 4A). A similar pattern was observed in terms of the strength of intercellular interactions (Fig. S5A). These results underscore the critical roles of NEAT1^+^ malignant cells and GFAP^+^ dSCs in PNI-positive dCCA. Subsequently, we utilized CellChat’s pattern recognition to identify major secretory signaling events of various cell types (Fig. 4B). When NEAT1^+^ malignant cells served as the signal source and GFAP^+^ dSCs as the signal input, the CDF15-TGFBR2 interaction exhibited the highest interaction score (Fig. 4B). Previous studies have demonstrated that inactivation of the *TGFBR2* gene leads to uneven and severely underdeveloped dSC invasion in mice (in vivo), hindering their involvement in the bridge regeneration process after nerve injury [39]. We also noted that the *CDF15* gene was predominantly expressed in NEAT1^+^ malignant cells, while *TGFBR2* was generally distributed across all SC types (Fig. 4C). Conversely, when GFAP^+^ dSCs sent ligands to NEAT1^+^ malignant cells, the primary interaction occurred through the BTC-EGFR pathway. The role of EGFR in cancer progression and as a therapeutic target in various human malignancies, including cholangiocarcinoma, lung cancer, colon cancer, and breast cancer, has been well-established [40–43]. Analyzing the receptor-ligand expression distribution, we found that BTC was almost exclusively expressed in GFAP^+^ dSCs, while EGFR was predominantly expressed by NEAT1^+^ malignant cells. Therefore, the BTC-EGFR interaction pair might represent a characteristic mode of communication between GFAP^+^ dSCs and NEAT1^+^ malignant cells (Fig. 4B-D).

To investigate the significance of the NEAT1^+^ malignant cell-GFAP^+^ dSC interaction within the TME, we utilized SCENIC [44] to decipher the gene regulatory network (GRN) of these cell types. The GRN differed among the subtypes of both malignant cells and SCs (Fig. S5B, C). We identified four key genes in the GRN of NEAT1^+^ malignant cells: *SREBF2*, *ATF3*, *RFX2*, and *JUN* (Fig. S5B). These genes have previously been shown to be upregulated in damaged neurons and regulate oxidative stress during the dedifferentiation of neighboring SCs [45, 46]. *JUN* is known to control mSC dedifferentiation and the activation of repair programs [47]. Conversely, multiple oncogenic transcription factors (TFs), including *ETS1*, *EP300*, *SMAD4*, and *ELK4*, were upregulated in GFAP^+^ dSCs (Fig. S5C). Jin et al. reported that tumor-derived extracellular vesicles promote renal cell carcinoma invasion and metastasis by transferring *MALAT1* facilitating the binding of *ETS1* and the *TFCP2L1* promoter [48]. Interestingly, *MALAT1* is one of the genes that characterize NEAT1^+^ malignant cells. In conclusion, our data highlight the close communication between NEAT1^+^ malignant cells and GFAP^+^ dSCs within the PNI-associated dCCA microenvironment and identify potential TF candidates for further investigation.

### Hypoxia induces lactate secretion from cancer cells and further promotes SC dedifferentiation

**Fig. 5:**
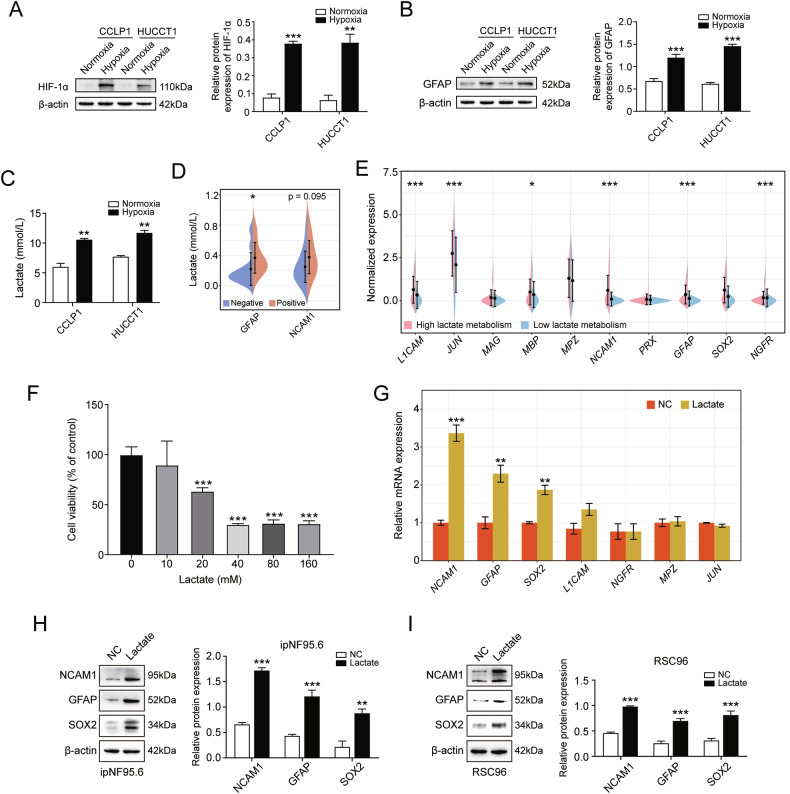
Hypoxia condition induced lactate secretion of CCA cells, further facilitated SC dedifferentiation. **A** The protein levels of HIF-1α in CCLP1 and HUCCT1 cells under normoxia and hypoxia conditions. **B** The protein levels of GFAP in ipNF95.6 cells receiving supernatants from CCA cells under normoxia and hypoxia conditions. **C** Detection of lactate in the supernatants of CCLP1 and HUCCT1 cells by lactate assay kit. **D** Detection of lactate in positive and negative nerve tissues for SC dedifferentiation markers (GFAP and NCAM1) in the Zhengzhou-dCCA cohort by lactate assay kit. **E** Expression levels of 10 dedifferentiation-related markers in SCs with high and low lactate metabolism. **F** The cytotoxic activity of lactate was measured using the MTT cell viability assay in ipNF95.6 cells. **G** Detection of mRNA expression levels of seven dedifferentiation-related markers in ipNF95.6 cells by RT‑qPCR. **H**, **I** Western blotting assays detecting protein levels of SC dedifferentiation members in ipNF95.6 (**H**) and RSC96 cells (**I**). *P < 0.05, **P < 0.01, ***P < 0.001.

Previous research demonstrated that pancreatic cancer cell supernatants under hypoxic conditions can induce GFAP activation in human SCs [49]. Of note, hypoxia is also a predicted hallmark of NEAT1^+^ malignant cells. To investigate the mechanism of SC dedifferentiation induced by hypoxic cells, we initially cultured CCLP1 and HUCCT1 cell lines under hypoxic conditions in vitro to simulate the in vivo hypoxic TME. After a 48-h incubation under either normoxic or hypoxic conditions, HIF-1α levels were detected via western blot analysis to assess the successful induction of hypoxic stress in the cancer cells. The results indicated a significant enhancement of HIF-1α expression under hypoxic conditions (Fig. 5A), confirming the effectiveness of our hypoxia modeling. Subsequently, we stimulated ipNF95.6 (a human SC line) with the modeled CCA cell supernatants to evaluate the activation of SCs by the hypoxic microenvironment of cancer cells. A significant increase in GFAP protein expression was observed when ipNF95.6 cells were exposed to the supernatant of the hypoxia group (Fig. 5B). Given that cancer cells consume substantial amounts of oxygen and nutrients, secreting excess lactate [50], and the high lactate metabolic activity of dSCs described above, we sought to determine whether SC dedifferentiation was related to lactate within the hypoxic TME. We first measured lactate levels in the supernatant of hypoxic cancer cells. Our findings revealed a significant elevation of lactate levels within the supernatant of hypoxic CCA cells (Fig. 5C). Similarly, we measured lactate levels in 22 dCCA tissues from the Zhengzhou-dCCA cohort, which were significantly higher in the GFAP protein-positive nerve group compared to the protein-negative group (Fig. 5D). For NCAM1 protein, there was a trend towards higher lactate content in the NCAM1 protein-positive nerve group, although these results were not statistically significant (Fig. 5D). Furthermore, we categorized all SCs from our scRNA-seq data into hypermetabolism and hypometabolism groups according to the median lactate metabolic activity score. We found that the expression of multiple dedifferentiation-related SC markers, including *L1CAM*, *JUN*, *NCAM1*, *GFAP*, and *NGFR*, was increased in the lactate hypermetabolism group (Fig. 5E). To examine the impact of lactate on SC dedifferentiation, we conducted a series of experiments. Referencing a previous study [51], we established a gradient lactate concentration (0, 10, 20, 40, 80, and 160 mM) to determine the optimal lactate concentration. SCs exposed to different lactate levels were cultured for 8 h, and their viability was assessed. The results indicated a dramatic decrease in SC viability at a lactate concentration of 20 mM. Consequently, we selected a lactate concentration of 10 mM for subsequent experiments (Fig. 5F). Next, we analyzed multiple representative dedifferentiation-related genes, among which the mRNA and protein levels of *NCMA1*, *GFAP*, and *SOX2* were noticeably upregulated in lactate-treated SCs (Fig. 5G-I). Additionally, we obtained cross-species validation in RSC96 (a rat SC line, Fig. 5I), indicating that the evolutionary process of lactate-induced dedifferentiation in SCs might be conserved. Collectively, these findings indicate that lactate produced by hypoxic cancer cells promotes the dedifferentiation of SCs.

### Cancer cell-derived lactate upregulates HMGB1 in SCs, which further promotes the carcinogenic behavior of CCA cells

**Fig. 6:**
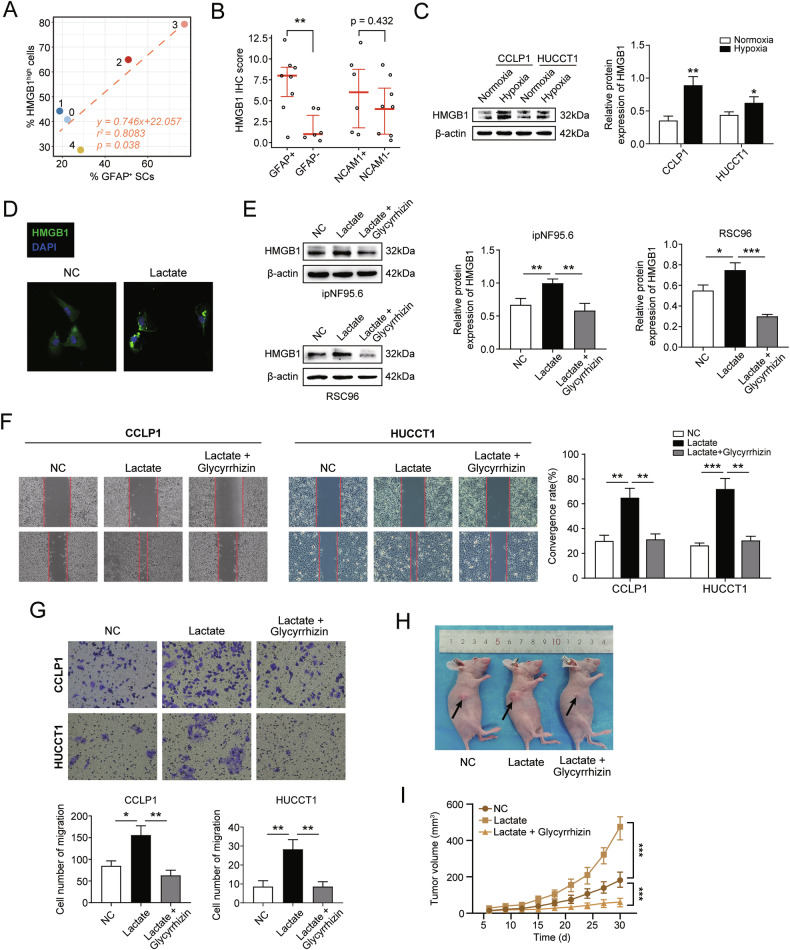
Cancer cell-derived lactate upregulated HMGB1 in SCs and HMGB1 further promoted carcinogenic behaviors in CCA cells. **A** The percentage of GFAP-positive cells is positively correlated with the proportion of cells with high levels of HMGB1. **B** The correlation between IHC expression of SC dedifferentiation markers (GFAP and NCAM1) and IHC scores of HMGB1 in the Zhengzhou-dCCA cohort. **C** The protein level of HMGB1 in ipNF95.6 cells receiving supernatants from cancer cells under normoxia and hypoxia conditions. **D** The observation of HMGB1 protein levels in ipNF95.6 cells using immunofluorescence. **E** The protein levels of HMGB1 in CCLP1 and HUCCT1 cells. **F**, **G** Wound healing assay (**F**) and trans-well invasion assay (**G**) were performed respectively to assess the mobility and invasion of cancer cells. **H** The xenograft tumor model was established with a mixture of CCLP1 cells and ipNF95.6 cells. The arrows indicate the subcutaneous tumor. **I** Tumor volumes were documented every 3 days. *P < 0.05, **P < 0.01, ***P < 0.001.

HMGB1 was initially reported to be released from lipopolysaccharide-stimulated macrophages and to function as a pro-inflammatory factor in sepsis [52]. More recent studies have demonstrated that stromal cells, such as tumor-associated macrophages, upregulate intracellular HMGB1 expression upon lactate stimulation, thereby promoting cancer progression [53, 54]. Interestingly, our single-cell data revealed that HMGB1 was generally upregulated in GFAP^+^ dSCs, and the percentage of GFAP^+^ cells within each SC subcluster correlated positively with the percentage of HMGB1^high^ SCs in that cluster. (Fig. 6A, Fig. S6A). Furthermore, a protein-protein interaction (PPI) network was constructed utilizing the STRING database v12.0, linking HMGB1 with 14 SC dedifferentiation-related genes. The PPI network indicated an interaction between HMGB1 and dSC markers, such as GFAP and JUN (Fig. S6B). To further examine the relationship between SC dedifferentiation and HMGB1 expression, we performed IHC in the Zhengzhou-dCCA cohort (Fig. S6C). The images revealed higher HMGB1 IHC scores in the neural tissues of GFAP and NCAM1 protein-positive groups were higher than those of protein-negative groups, although the latter showed no statistical significance (Fig. 6B, Table S7). Additionally, we found that HMGB1 protein was significantly upregulated after stimulation of ipNF95.6 cells with cancer cell supernatants after hypoxia incubation (Fig. 6C). To determine if HMGB1 expression in SCs was similarly linked to lactate secreted in the hypoxic TME, immunofluorescence (IF) experiments demonstrated an increase in the cytoplasmic level of HMGB1 in lactate-treated ipNF95.6 cells (Fig. 6D). To investigate the role of HMGB1 in SC dedifferentiation and its impact on tumor progression, we introduced glycyrrhizin (1 nM) to inhibit HMGB1 expression in subsequent protein immunoblotting and IF experiments [55]. Our findings revealed a significant elevation of HMGB1 protein levels in lactate-stimulated SCs, which was effectively inhibited by glycyrrhizin (Fig. 6E, Fig. S6D). Next, we sought to understand whether SCs stimulated with lactate promoted tumor progression through HMGB1. Co-culture experiments with lactate-induced SCs demonstrated accelerated cell migration and invasion in both CCA cell lines (Fig. 6F, G). Nevertheless, glycyrrhizin reversed the lactate-induced effect (Fig. 6F, G). To further evaluate the functional role of lactate-treated dSCs in CCA progression, we conducted in vivo experiments using xenograft mice. Mice injected with a mixture of lactate-stimulated SCs and CCLP1 cells exhibited larger tumor volumes. Notably, glycyrrhizin attenuated the tumor growth-promoting effect of lactate-stimulated SCs through HMGB1 inhibition (Fig. 6H, I). Considering that the nuclear protein HMGB1 is released in response to diverse stimuli, including lactate [56, 57], we focused on the expression level of HMGB1 within tumor cells after co-culture with SCs. Lactate-treated SCs elevated the level of HMGB1 within cancer cells, while glycyrrhizin inhibited this elevation (Fig. S6E). Collectively, these results suggest that SCs enhance the invasion and migration of cancer cells through lactate-induced upregulation of HMGB1.

## Discussion

At present, dCCA, a subtype of CCA, remains a highly lethal disease despite significant advancements in scientific understanding and clinical management [2]. In recent years, numerous studies have been conducted on the molecular pathogenesis of CCA especially for iCCA. Yet the unraveling of the molecular complexity of dCCA remains limited, and there are no approved targeted therapies with demonstrated clinical benefit. PNI, a common pathological feature in dCCA, is strongly associated with postoperative recurrence and poor prognosis [58]. Several studies have highlighted the pivotal role of dSCs in PNI and cancer progression [10, 59, 60]. A deep appreciation for the cellular ecosystem of PNI-associated dCCA and the potential molecular mechanisms underlying the contribution of dSCs to PNI remains an unmet clinical need. In our study, we utilized scRNA-seq to comprehensively map the transcriptomic landscape of human PNI-positive dCCA, unveiling novel cell-cell communications between dCCA cells and dSCs at single-cell resolution.

Through scRNA-seq analysis, we identified multiple distinct cell types within PNI-positive dCCAs. Lymphoid cells predominated in PNI-positive dCCAs, accounting for over 30% of all cells, followed by myeloid cells and epithelial cells (both malignant and normal epithelium). The distribution of individual cell subsets within a single sample varied significantly, demonstrating substantial inter-tumor heterogeneity. scRNA-seq profiling enabled the definition of three distinct malignant subtypes. These three malignant subtypes exhibited specific differentially expressed genes (DEGs) and potential TFs. Importantly, we observed that an accumulation of NEAT1^+^ malignant cells was related to poorer clinical outcomes and the development of PNI in dCCA patients, suggesting their highly malignant properties. Notably, we found that NEAT1^+^ malignant cells displayed a highly hypoxic profile. Hypoxia-induced NEAT1 has been reported to be mediated by HIF-2α transcriptional activity [61, 62]. CNV-wise analysis revealed a significantly higher proportion of CNV-high malignant cells within NEAT1^+^ malignant cells than other malignant cell types, similarly confirming a malignant nature. Our research employed pseudotemporal trajectory analysis to identify distinct states of the three malignant cells and further characterize their developmental dynamics. Among these states, NEAT1^+^ malignant cells might represent an earlier stage of differentiation in dCCA cells. We identified several NEAT1^+^ malignant cell-associated genes, many of which are related to hypoxic and oncogenic signaling pathways, such as *VEGFA*, *AKT*, *JUN*, and *KRAS*. Previous studies have reported that *JUN* promotes de-differentiation of SCs after neural injury by inhibiting *P0*, *MBP*, and *KROX20* [37, 63]. Interestingly, *JUN* was also a predicted regulator of NEAT1^+^ malignant cells. This suggests the possibility of *JUN*-mediated cellular communication between these malignant cells and dSCs, which warrants further investigation. Additionally, upregulated MALAT1 in NEAT1^+^ malignant cells was associated with synapse formation and neuronal cell survival [64], potentially resulting from the close interaction between NEAT1^+^ malignant cells and dSCs predicted at the single-cell level. In conclusion, our findings highlight that NEAT1^+^ malignant cells may represent a class of malignant cells with hypoxia propensity that dominate the PNI-positive dCCA tissues.

As major constituent cells of nerves, SCs have been demonstrated to promote tumor growth and play a pivotal role in PNI across multiple tumor types. In this study, we provided hitherto undocumented evidence of five distinct SC subtypes in human dCCA and its adjacent tissues. Notably, nmSCs and GFAP^+^ dSCs exhibited a higher proportion of cells within dCCA tissues, while SCPs, PMP22^+^ mSCs, and MPZ^+^ mSCs were predominantly found in the adjacent tissues, highlighting the heterogeneity of the neurological tissue microenvironment in dCCA. Our analysis revealed that GFAP^+^ dSCs expressed minimal myelinating but high levels of immature SC genes, suggesting they might be a biochemically and metabolically active subpopulation of SCs. Subsequently, we verified that CCA cell-derived lactate is a metabolite that induces and maintains GFAP^+^ dSC dedifferentiation. It has been reported that monocarboxylate transporter protein (MCT) is highly expressed in perineuronal cells and facilitates lactate uptake as its preferred energy metabolite [65, 66]. The dependence of peripheral nerve function on lactate metabolism was further emphasized in a study by Morrison *et al*., where MCT1 deficiency impeded nerve regeneration after peripheral nerve injury in mice [67]. Importantly, survival analysis and pseudotemporal analysis indicated that GFAP^+^ dSCs may represent a harmful SC population within the PNI-positive dCCA neural microenvironment, potentially originating from MPZ^+^ mSCs. Therefore, the GFAP^+^ dSCs subpopulation may serve as a promising therapeutic target for dCCA patients with concomitant PNI.

HMGB1, a representative injury-associated molecule, has been implicated in various pathological processes, including neurodegenerative diseases, autoimmunity, and cancer progression [68, 69]. Peripheral nerve injury can induce HMGB1 expression through the proliferation of SCs and infiltration of macrophages within nerves [70, 71]. HMGB1 expression is significantly elevated in pCCA tissues [72] and is associated with poor prognosis, lymphatic invasion, and direct involvement in CCA proliferation and angiogenesis [72, 73]. Recent studies have demonstrated that lactate stimulates macrophage M2 polarization and secretes HMGB1, thereby promoting glioma cell invasion [74]. Our IHC staining has confirmed that GFAP^+^ and NCAM1^+^ peripheral nerves express high levels of HMGB1 protein internally. This prompted us to investigate whether SCs also act as a lactate-induced “HMGB1 reservoir”, contributing to neural infiltration by dCCA cells. In this study, we found that CCA cell-derived lactate stimulated the dedifferentiation of SCs and significantly induced HMGB1 expression in GFAP^+^ dSCs, enhancing malignancy of cancer cells. It is widely believed that HMGB1 may directly contribute to tumor cell metastasis by modifying extracellular matrix components and regulating cell adhesion properties [75] or enhance tumor cell progression by inducing MIA [76]. Cellular immunofluorescence confirmed a significant upregulation of HMGB1 in CCA cells following co-culture with SCs that exocrine HMGB1, suggesting its potential role in exacerbating the oncogenicity of cancer cells.

To sum up, our study presents a uniquely matched set of transcriptomic landscapes within the TME of PNI-positive dCCA and paracancerous samples, offering a valuable resource for elucidating SC diversity in PNI-positive dCCA. This study also highlights the intra-tumor crosstalk between PNI-associated malignant cells and dSCs. Future research is warranted to corroborate the molecular mechanisms underlying dCCA PNI, and our dataset can serve as a valuable tool for designing targeted therapeutics against PNI-positive tumors.

## Methods

### Patients and clinical samples collection

Two patients with dCCA who did not receive preoperative chemotherapy or radiotherapy participated in this study. Informed consent was obtained from all participants, who were requested to donate their tumor tissues and corresponding peri-tumor tissues for scientific research. Tissue samples were transported on ice and processed within 30 min of acquisition.

### scRNA-seq and data analysis of dCCA tissues

Single-cell suspensions were prepared for each sample. Cell viability was ensured to be above 70%, and the cell concentration was adjusted to 300–600 cells/μL. scRNA-seq was performed using the 10× Genomics Chromium Single Cell 3’ platform following the manufacturer’s instructions. The generated count matrices were converted to a Seurat object using the Seurat package (version 4.4.0) [77]. Cells expressing fewer than 200 genes or with mitochondrial reads exceeding 40% were excluded from downstream analysis. Batch effect correction was conducted using the Harmony package [78], and the filtered gene-barcode matrices were normalized using the LogNormalize method. The top 3000 highly variable genes for principal component (PC) analysis were identified using the FindVariableFeatures function. The top 30 PCs were then selected for uniform manifold approximation and projection (UMAP) visualization of the cells. For cell clustering, the FindClusters function was employed at a resolution of 0.3. Subgroup cell clusters were analyzed by selecting the top 30 PCs and clustering at various resolutions, which were determined through visual inspection.

### Distinguish malignant and non-malignant epithelial cells based on inferred CNVs

Initial CNVs were estimated using the inferCNV package (version 1.12.0), as previously described [79]. To minimize the impact of genes with extreme expression, the expression values were re-standardized and restricted to the range [−2,2]. For each cell, the mean of the squared CNV*i* (CNV of the *i*th window) across the genome was calculated as the CNV signal. Additionally, the CNV correlation values were calculated by correlating the CNV profile of a single cell with the average CNV*i* profile of the top 5% of cells with the highest CNV scores. Epithelial cells with a CNV signal above 0.225 and a CNV correlation above 0.45 were classified as malignant.

### Identification of high-confidence hypoxic cells using the CHPF model

CHPF [26] is an open-source modeling framework designed to predict cellular hypoxia status. The CHPF script was executed in Python (version 3.11.5). The single-cell expression profile and seven pre-selected hypoxia-related gene sets served as input files for the construction of the prediction model. The final formula was provided below:

$$P(x)=\frac{{\sum }_{i=1}^{n}{W}_{i}(x){f}_{i}(x)}{{\sum }_{i=1}^{n}{W}_{i}(x)}(n=100)$$

Cells with *P*(*x*) > 0.5 were considered as hypoxic cells.

### Pseudotime analysis by Monocle

Pseudo-time analysis and transcriptome dynamic analysis along the pseudo-time trajectory were conducted using Monocle2 (version 2.26.0) [80] with the default parameters recommended by the developer.

### Deconvolution analysis

We employed CIBERSORTx (https://cibersortx.stanford.edu/), a deconvolution analysis tool, to investigate gene expression within the TME. Our analysis focused on the 10x scRNA-seq data, specifically looking for DEGs between epithelial cell subtypes and SC subtypes. These genes were used to create a signature matrix. To deconvolute the bulk RNA-sequencing data, we employed two separate reference sources: 1) data from the TCGA-CHOL cohort within The Cancer Genome Atlas (TCGA); and 2) an RNA-sequencing dataset (GSE107943) downloaded from the Gene Expression Omnibus database. These datasets served as the mixture files for CIBERSORTx analysis.

### H&E staining and IHC assay

22 cases of dCCA tissues that underwent pancreaticoduodenectomy were obtained from the First Affiliated Hospital of Zhengzhou University between 2021 and 2024. Tissues were fixed with 4% paraformaldehyde, embedded in paraffin, and cut into 4-μm thick sections.

For H&E staining, the sections were stained with hematoxylin (BA4097, BaSo Diagnostics Inc., Zhuhai, China) for 5 min and eosin (BA4098, BaSo Diagnostics Inc.) for 3 min. For the IHC assay, sections were deparaffinized, rehydrated, and blocked. Primary antibodies were incubated at 4 °C overnight, followed by incubation with goat anti-mouse IgG H&L HRP (1:4000, SA00001-1, Proteintech, Wuhan, China) at room temperature for 2 h. The primary antibodies used in this study included GFAP (1:5000, 60190-1-Ig, Proteintech), NCAM1 (1:5000, 14255-1-AP, Proteintech), and HMGB1 (1:400, 66525-1-Ig, Proteintech). The staining extent score (<25%, score = 1; 25–50%, score = 2; 50–75%, score = 3; >75%, score = 4) and staining intensity (negative, score = 0; weak, score = 1; moderate, score = 2; strong, score = 3) were assessed using ImageJ software 1.46r. IHC results were scored by multiplying the staining extent score by the intensity score. All H&E stained and IHC sections were scanned with a Pannoramic MIDI II scanner (3D HISTECH Ltd., Hungary).

### Cell culture and treatment

CCLP1 (JNO-H0653) and HUCCT1 (BNCC337995) human CCA cell lines were obtained from Jennio Biotech Co., Ltd. (Guangzhou, China) and Beina Chuanglian Biotechnology Institute (Beijing, China), respectively. Human ipNF95.6 SCs (CTCC-001-0379, Meisen CTCC, Panan, China) and rat RSC96 SCs (CL-0199, Pricella Biotechnology Co., Ltd., Wuhan, China) were maintained in our laboratory. All cells were cultured in DMEM (12100, Solarbio, Beijing, China) medium supplemented with 10% fetal bovine serum (C04001-500, VivaCell, Shanghai, China). The normoxia group cells were cultivated at 37 °C in a 5% CO_2_ humidified incubator (Galaxy 170R, Eppendorf, Hamburg, Germany). For hypoxia induction, cells were transferred to a tri-gas incubator (Galaxy 48R, Eppendorf) and incubated under 1% O_2_, 5% CO_2_, and 94% N_2_ for 48 h prior to commencing subsequent experiments.

A non-contact coculture system was established using 24-well plates containing 0.4-μm polyethylene terephthalate membrane filters (Corning, NY, USA) to separate the lower and upper chambers. ipNF95.6 cells, subjected to various treatments, were seeded in the upper chamber at a density of 1 × 10^5^ cells/mL. CCLP1 or HUCCT1 cells were then inoculated in the lower chamber at a density of 1.5 × 10^5 ^cells/mL. Following a 48-h incubation period, CCA cells were harvested for subsequent experiments.

To stimulate lactate production, SCs were cultured for 8 h with increasing concentrations of lactate (0, 10, 20, 40, 80, and 160 mM; L1750, Merck, NJ, USA).

Glycyrrhizin (B20417-20mg, Yuanye Bio-Technology, Shanghai, China), a direct inhibitor of HMGB1, was added to SCs [55] at a concentration of 1 nM in conjunction with lactate to suppress HMGB1 expression in these cells.

The supernatants of CCA cells cultured under normoxic or hypoxic conditions were collected by centrifuging at 500 × *g* for 10 min, followed by a second centrifugation at 2000 × *g* for 20 min. After any necessary pre-treatments, SC supernatants were collected using the same procedure. These collected supernatants were stored at −80 °C and used within one month.

### Cell–cell interaction analysis

Intercellular communication between malignant and SC types was investigated using the CellChat package (version 1.6.1) [81]. The Seurat-normalized data was transformed into a CellChat object using the createCellChat function. Subsequently, the computeCommunProbPathway function was employed to infer intercellular communication at a signaling pathway level.

### SCENIC analysis

SCENIC (version 1.3.1) was utilized to evaluate the transcriptional activity of malignant cells and SCs [44]. SCENIC was implemented in R using the motif databases of RcisTarget and GRNboost (corresponding to GENIE3 1.20.0, AUCell 1.22.0, and RcisTarget 1.18.2). Raw UMI counts served as input for the analysis.

### Western blot assay

The total proteins were extracted with RIPA buffer (R0010, Solarbio). Proteins were separated by 10% SDS-PAGE and transferred to nitrocellulose membranes. The membranes were incubated with primary antibodies at 4 °C overnight, and specific binding of the primary antibodies was detected with peroxidase-labeled goat anti-mouse (1:4000, SA00001-1, Proteintech) or goat anti-rabbit (1:4000, SA00001-2, Proteintech) secondary antibodies. The following primary antibodies were used: HIF-1α (1:4000, ab51608, Abcam), GFAP (1:5000, 60190-1-Ig, Proteintech), NCAM1 (1:20,000, 14255-1-AP, Proteintech), SOX2 (1:1000, #2748, CST, Boston, USA) and HMGB1 (1:3000, 66525-1-Ig, Proteintech) and β-actin (1:40,000, 66009-1-Ig, Proteintech).

### Lactate measurement

The lactate concentrations in cell supernatants and tissues were determined by an L-Lactate Assay Kit (A019-2-1, Nanjing Jiancheng Bioengineering Institute, Nanjing, China). Samples were prepared according to the manufacturer’s instruction and the lactate levels of the samples were calculated by measuring the absorbance at 530 nm.

### Cell cytotoxicity experiment

For the thiazolyl blue tetrazolium bromide (MTT) assay, MTT (M5655, Sigma, Shanghai, China) was dissolved in DMEM at a concentration of 5 mg/mL. Cells were incubated with MTT for 4 h at 37 °C under 5% CO_2_. After removing the MTT, the formed MTT-formazan crystal was dissolved in DMSO (150 μL/well). Absorbance at 490 nm was measured using a microplate reader (Spark, Tecan, Switzerland). The results were expressed as the percentage change in absorbance compared to untreated control cells, which were set to 100%. Data represent the average of triplicate measurements from three independent experiments.

### Real‑time quantitative polymerase chain reaction (RT‑qPCR)

Total RNA from cells was extracted using TriQuick Reagent (R1100, Solarbio). cDNA was synthesized using NovoScriptPlus All-in-one 1st Strand cDNA Synthesis SuperMix (E047-01B, Novoprotein Scientific Inc., Shanghai, China). RT-qPCR was conducted in a 20-μL reaction volume containing forward and reverse primers, cDNA, and NovoStart SYBR qPCR SuperMix Plus (E096-01A, Novoprotein Scientific Inc.). All primers were synthesized by Sangon Biotech and normalized to GAPDH. RNA folding changes were quantified using the 2^−ΔΔCt^ method. The primer sequences are listed in Table S8.

### Immunofluorescence staining

Cell climbing slices were sterilized with 75% alcohol, air-dried in 24-well plates, and inoculated with CCA cells or SCs. After that, cells were fixed with 4% paraformaldehyde, permeabilized with 0.2% Triton X-100 for 15 min at 4 °C, and blocked with 5% bovine serum albumin. Primary HMGB1 antibody (1:200, 66525-1-Ig, Proteintech) was added overnight at 4 °C. Cells were then incubated with goat anti-mouse IgG (H&L) secondary antibody (1:400, Alexa Fluor 488, GB25301, Servicebio, Wuhan, China) or (1:200, Alexa Fluor 594, AB0152, Abways, Shanghai, China) for 2 h at room temperature, protected from light, and counterstained with DAPI for 10 min. Images were captured using a DM4B microscope system (Leica, Wetzlar, Germany).

### Wound healing assay

A coculture system was employed for the scratching experiment. Following coculture completion, the upper chamber was removed, and the cells in the lower chamber were rinsed with phosphate-buffered saline (PBS). Cells were maintained in culture until reaching 90% confluence. Two lines were then scratched using a 200 μL pipette tip. Nonadherent cells were washed away twice with PBS and incubated in serum-free DMEM for 24 h. Microscopic observation and photography were performed at 0 and 24 h. The ImageJ software (version 1.6.0) was utilized to analyze the wound healing rate by quantifying the wound closure area.

### Cell invasion assay

A cell invasion assay was conducted using 24-well plates equipped with 8.0-μm pore polycarbonate membrane inserts (Corning). Firstly, 100 μL matrigel (diluted with the serum-free DMEM at 1:8, cat#354234, Corning) was added to the upper chamber and incubated at 37 °C for 2 h. Then, the upper chamber was added with 100 μL serum-free DMEM and incubated for another 30 min. Afterward, the liquid in the chamber was removed. A 200 µL cell suspension (serum-free DMEM) was seeded in the upper chamber, and Medium (600 µL) containing 10% FBS was added to the lower chamber. Following incubation at 37 °C for 48 h, non-migratory cells on the top surface of the inserts were gently removed with cotton swabs. Subsequently, the inserts were fixed in 4% paraformaldehyde for 15 min, and the cells were stained with 0.2% crystal violet. The number of cells that invaded the membrane was quantified under a light microscope.

### Tumorigenicity assay

Tumor xenografts were established in 5-week-old female BALB/c nude mice obtained from Beijing Vital River Laboratory Animal Technology Co., Ltd (Beijing, China). The mice were maintained in specific pathogen-free conditions with a 12-h light/dark cycle. All mice were randomly allocated to 3 groups (8 mice per group). After acclimatization for 1 week, 5 × 10^6^ CCLP1 cells mixed with 2 × 10^6^ ipNF95.6 cells were injected subcutaneously on the right flank of the mice. ipNF95.6 cells were pretreated with PBS, lactate, or lactate combined with glycyrrhizin for over three generations. Following inoculation, tumor volume was measured every three days and calculated using the formula *V* = *ab*^2^/2, where *a* is the long diameter and *b* is the short diameter.

### Statistical analysis

Data were analyzed using GraphPad Prism (version 8.0.1.244, GraphPad Software Inc., San Diego, USA). The Shapiro-Wilk test was used to assess the normality of each distribution. Student’s *t*-test or the Wilcoxon rank-sum test was employed to compare two groups, while ANOVA followed by Tukey’s multiple comparisons test was used for multiple group comparisons. Chi-square test was used for categorical variables distribution test. Kaplan-Meier curves were constructed and analyzed using the log-rank test. All data were presented as means ± standard deviation. All in vitro experiments were conducted in at least three independent experiments. *P < 0.05, **P < 0.01, ***P < 0.001, ns: not significant.

## Supplementary information

Supplemental Figures

Supplemental Tables

Raw WB data

## Data Availability

The raw data of scRNA-seq are available from W.Z. or Z.Z. on reasonable request. The codes supporting the results of this article are deposited in the Science Data Bank (www.scidb.cn), 10.57760/sciencedb.18768. CHPF is available online at https://github.com/yihan1221/CHPF. Publicly available datasets were analyzed in this study: datasets stored in TCGA for CCA at http://xena.ucsc.edu/ and GSE107943 at https://www.ncbi.nlm.nih.gov/geo/. All other datasets used and/or analyzed during the current study are available within the manuscript and its supplementary information file.
