# Supplementary material for: Single-cell analysis reveals that GFAP+ dedifferentiated Schwann cells promote tumor progress in PNI-positive distal cholangiocarcinoma via lactate/HMGB1 axis
Source: Cell Death Dis. 2025 Mar 27;16(1):215. doi: 10.1038/s41419-025-07543-x (PMC11950304; doi:10.1038/s41419-025-07543-x)
Supplement: Supplementary file 1 — Supplemental Figures [file 41419_2025_7543_MOESM1_ESM.docx]

**Supplementary Figures:**


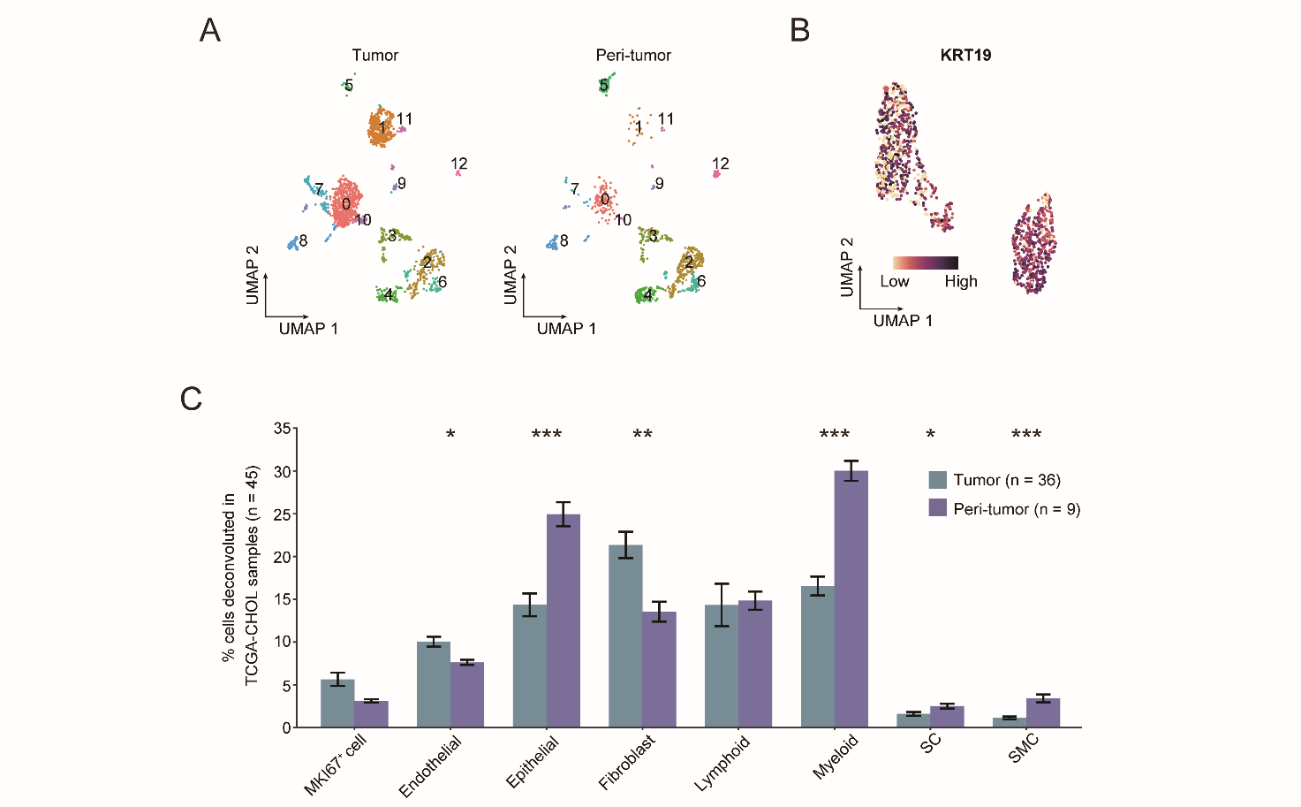


**Figure S1:** (A) UMAP plot of cells in tumor and peri-tumor tissues from 2 dCCA samples. (B) UMAP plot showing the expression of KRT19 in all the malignant cells. (C) Bar plot showing the cell type abundance for samples from different groups, as measured by deconvoluted bulk RNA-seq data from TCGA-CHOL cohort. **p* < 0.05, ***p* < 0.01, ****p* < 0.001.


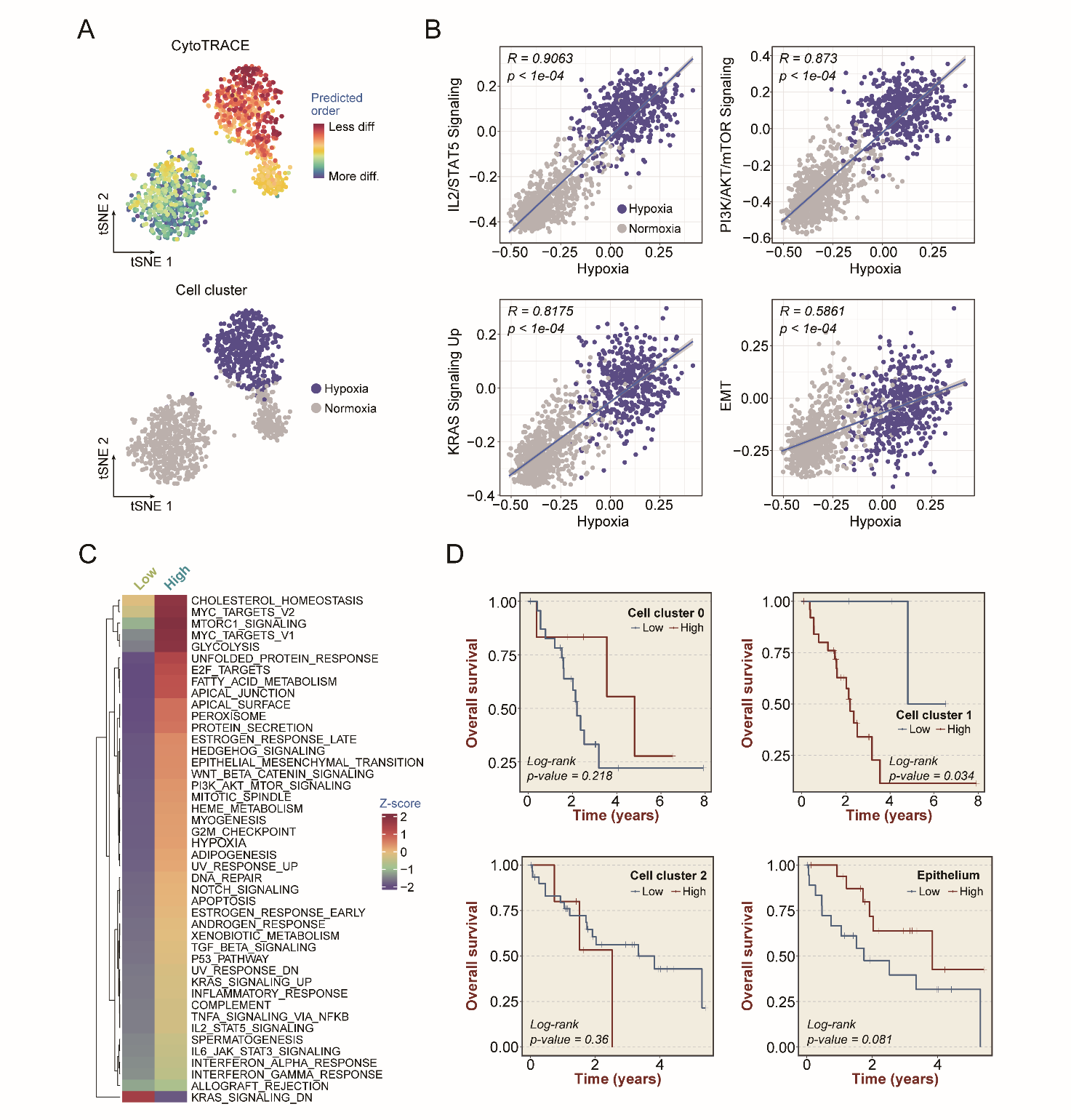


**Figure S2:** (A) tSNE plot (top) depicting the distribution of CytoTRACE scores among malignant cells. Dark-blue indicates lower scores (low stemness) while dark-red indicates higher scores (high stemness). tSNE plot (bottom) labelling the malignant cells by hypoxia status. (B) Correlation analysis of hypoxia with IL2/STAT5, PI3K/AKT/mTOR, KRAS UP (genes upregulated by KRAS activation), and EMT signalings. (C) Heatmap showing functional pathways activated in malignant cells with different CNV levels using GSVA analysis. (D) Kaplan–Meier curves of GSE107943 patients (n = 30) showing the survival rates grouped by the cell abundance in all the epithelial cell clusters.


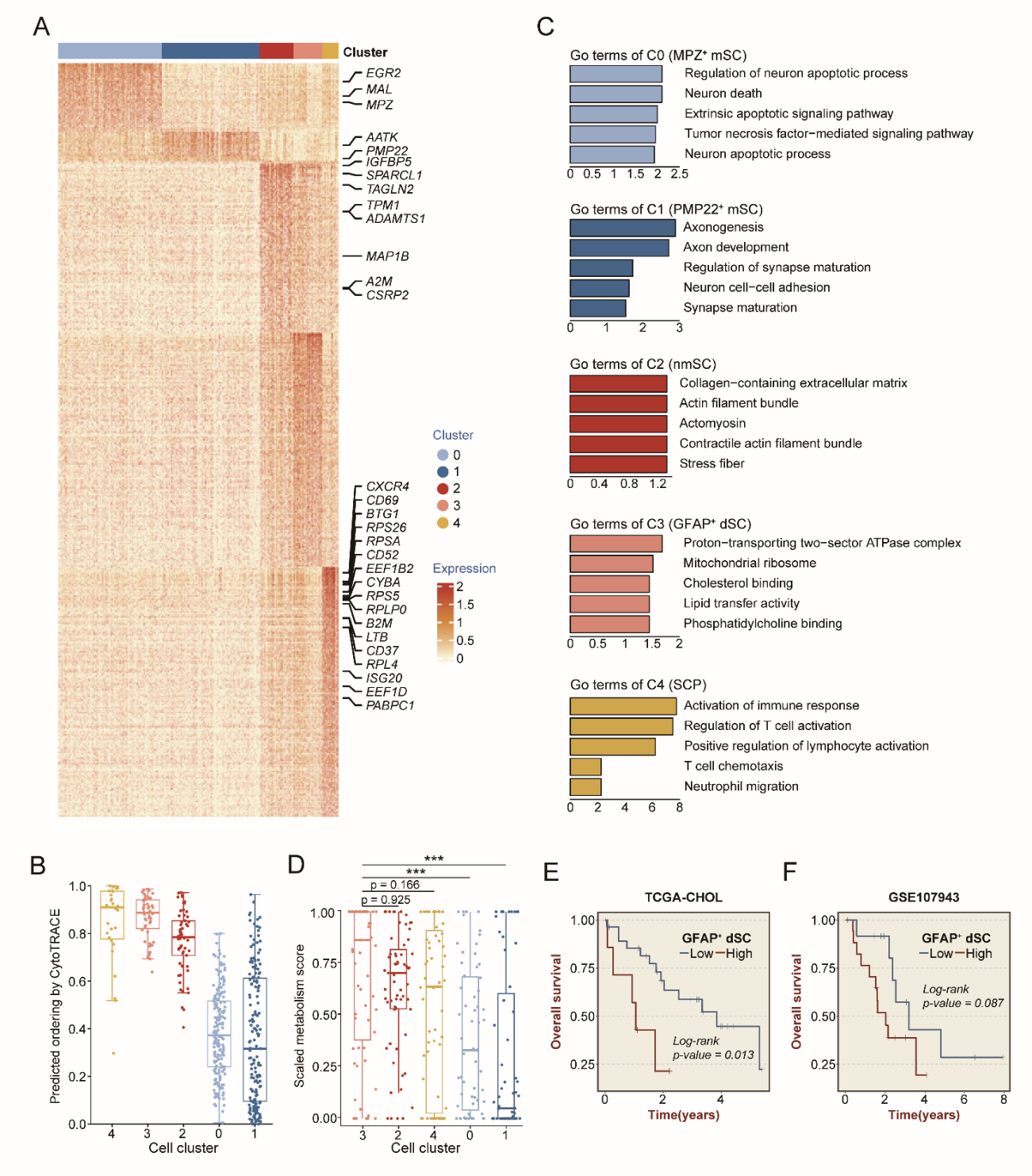


**Figure S3:** (A) Heatmap showing the scaled expression levels of SC subtype-specific marker genes. (B) CytoTRACE score of each SC subpopulation. (C) GO analysis of DEGs in distinct SC subclusters. (D) Boxplot showing the metabolic score of metabolic pathways in each SC subpopulation. (E) Kaplan–Meier curves of TCGA-CHOL patients (n = 36) showing the survival rates grouped by the cell abundance in GFAP^+^ dSCs. (F) Kaplan–Meier curves of GSE107943 patients (n = 30) showing the survival rates grouped by the cell abundance in GFAP^+^ dSCs. ****p* < 0.001.


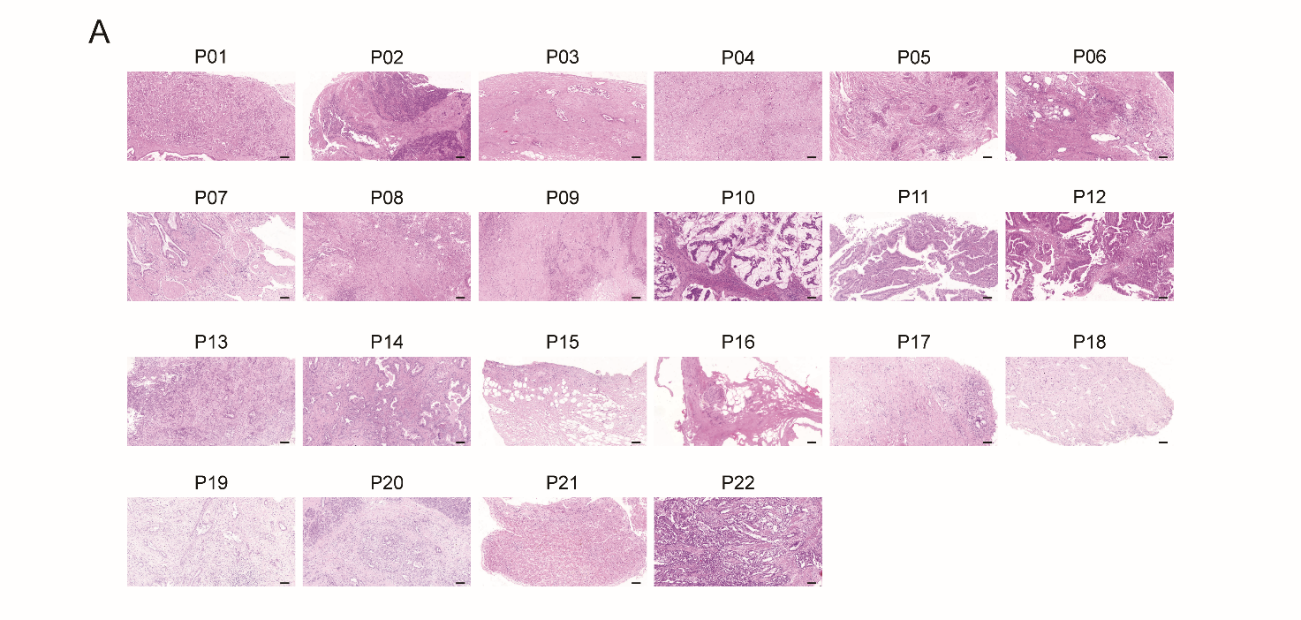


**Figure S4:** H&E staining of patients from the Zhengzhou-dCCA cohort (n = 22). The experiment was repeated once with similar results. Scale bars, 100 μm.


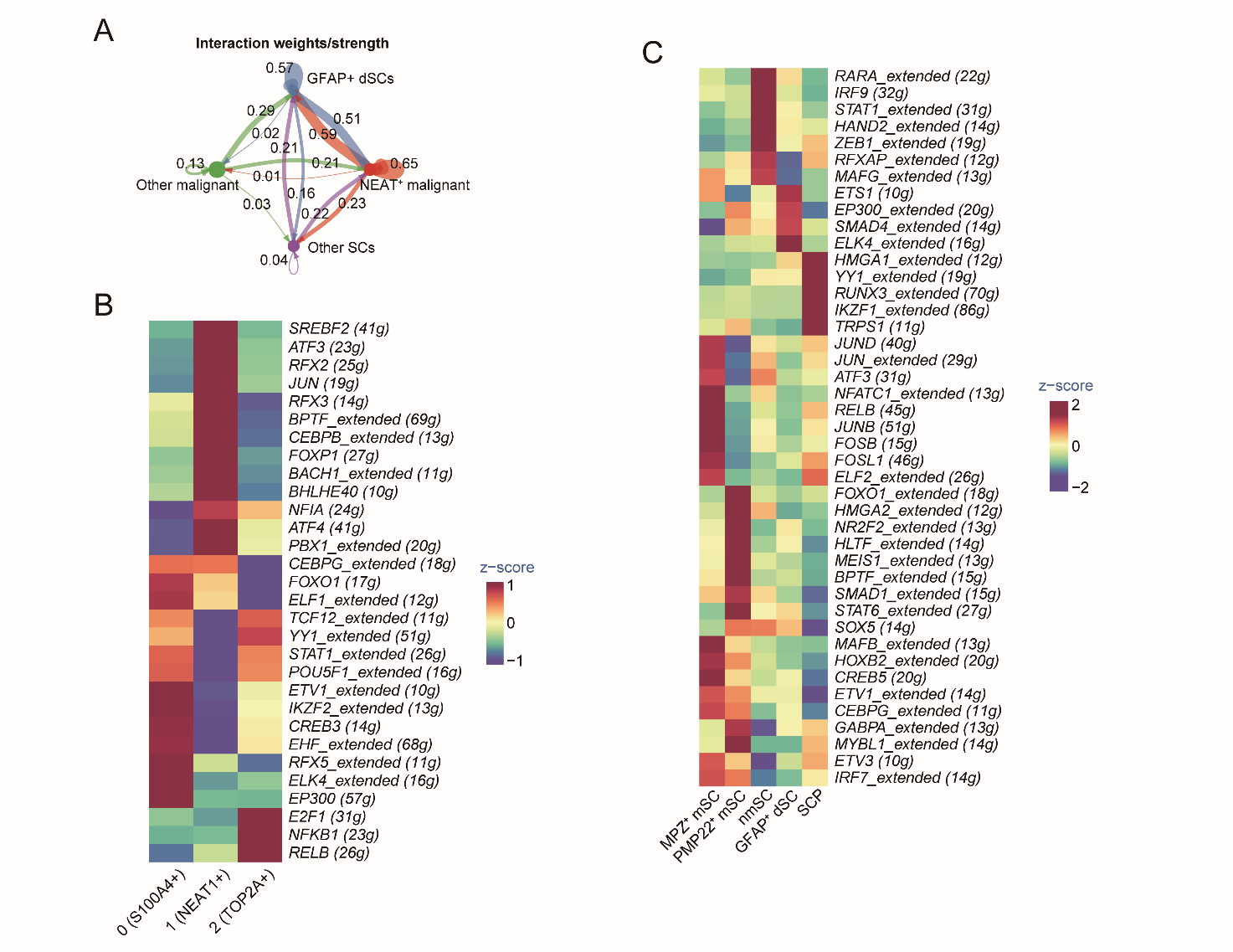


**Figure S5:** (A) Cell-cell interaction network of NEAT1^+^ malignant cells, other malignant cells, GFAP^+^ dSCs, and other SCs. The node sizes are proportional to the numbers of each cell type. The width of the edge and numbers represent the relative strength of significant ligand-receptor interactions in two cell types. Subpopulation-specific regulons of each malignant cell subpopulation (B) and SC subpopulation (C) using SCENIC analysis.


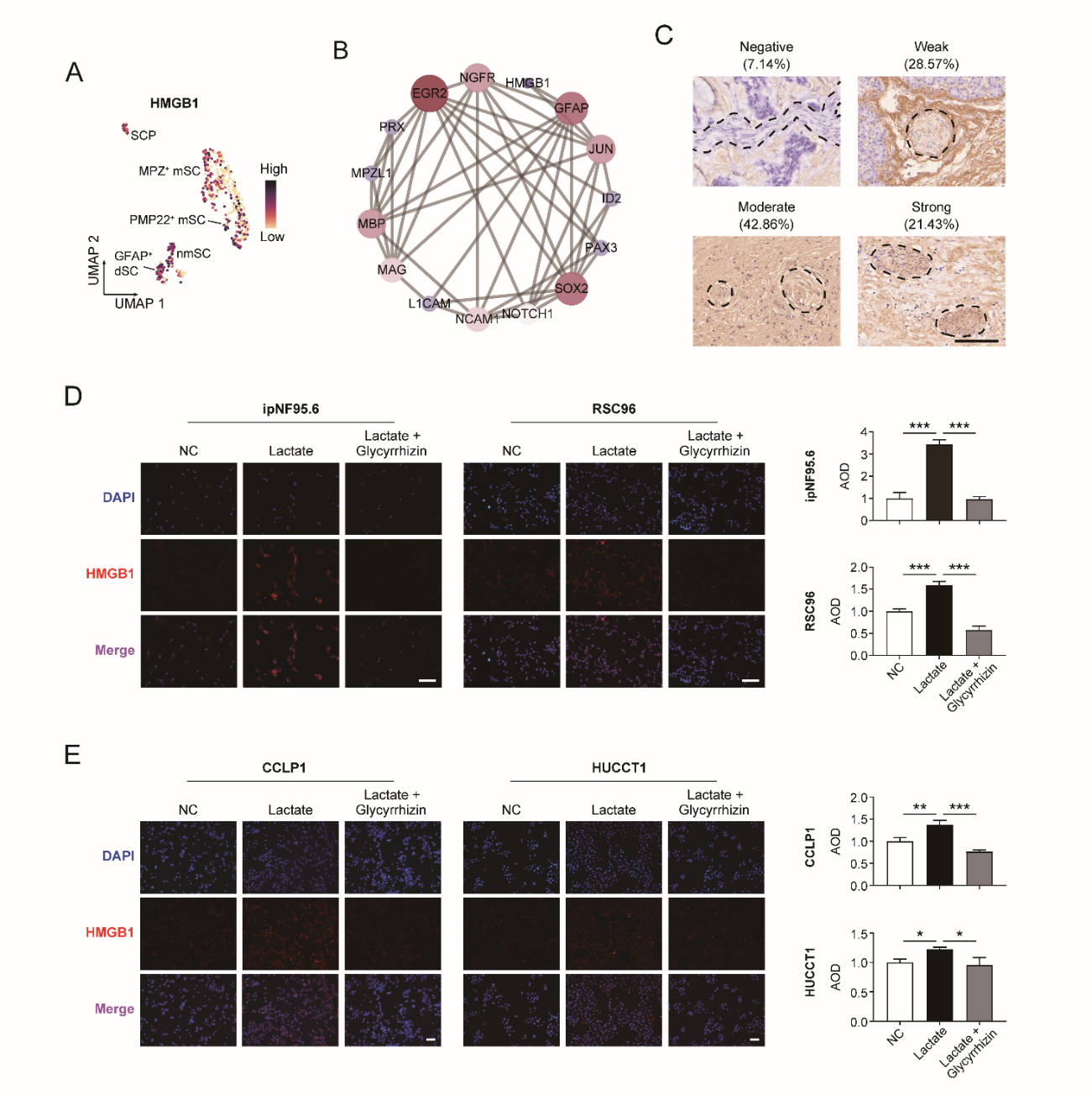


**Figure S6:** (A) UMAP plot showing the expression of HMGB1 in all the SCs. (B) Protein-protein interactions between HMGB1 and 14 dSC markers. (C) Representative IHC staining of HMGB1 protein in the Zhengzhou-dCCA cohort. The experiment was repeated once with similar results. Scale bar, 100 μm. (D) Representative IF images of HMGB1 protein levels in ipNF95.6 and RSC96 cells. The experiment was repeated once with similar results. Scale bars, 100 μm. (E) Representative IF images of HMGB1 protein levels in CCLP1 and HUCCT1 cells. The experiment was repeated once with similar results. Scale bars, 100 μm. **p* < 0.05, ***p* < 0.01, ****p* < 0.001.
