# Supplementary material for: Single-cell analysis reveals that GFAP+ dedifferentiated Schwann cells promote tumor progress in PNI-positive distal cholangiocarcinoma via lactate/HMGB1 axis
Source: Cell Death Dis. 2025 Mar 27;16(1):215. doi: 10.1038/s41419-025-07543-x (PMC11950304; doi:10.1038/s41419-025-07543-x)

Supplementary Information

Uncropped scan images used in this manuscript. Cropped imaged used are indicated by red squares

Figure 5A

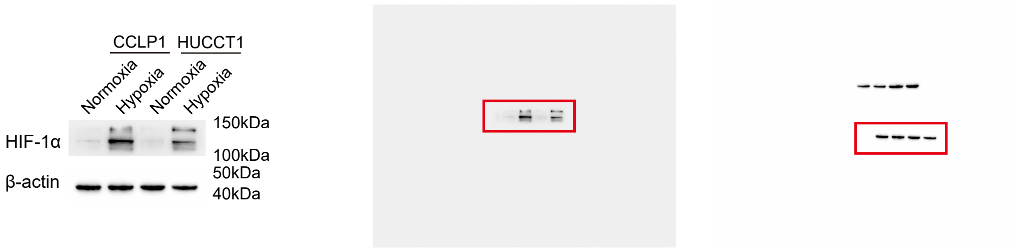

Figure 5B

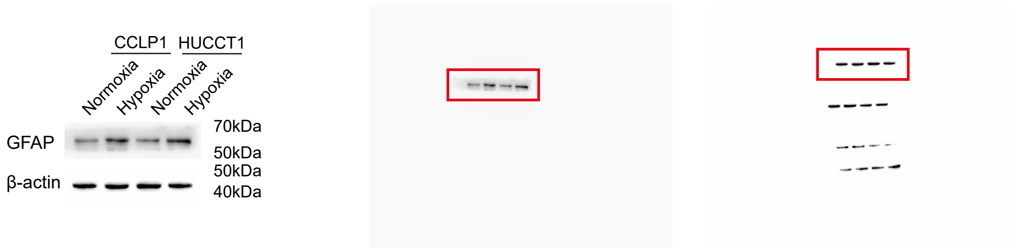

Figure 5H

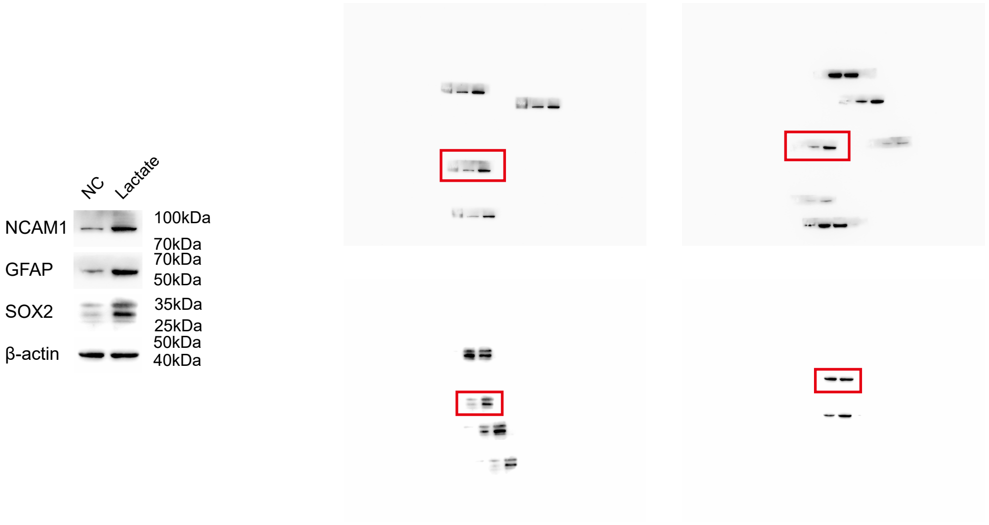

Figure 5I

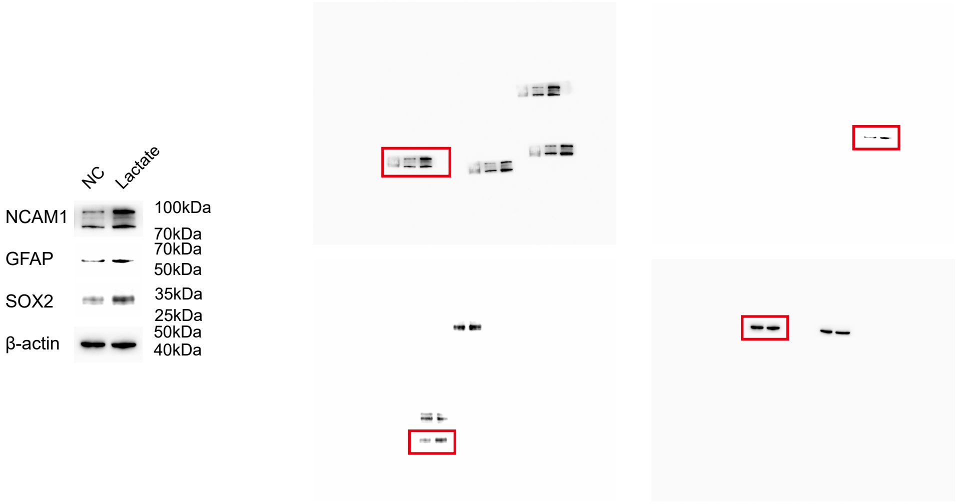

Figure 6C

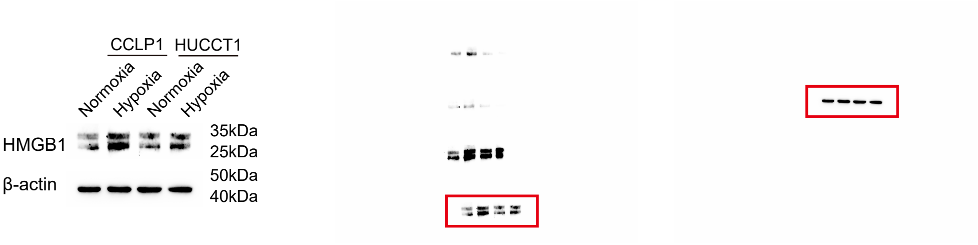

Figure 6E (ipNF95.6)

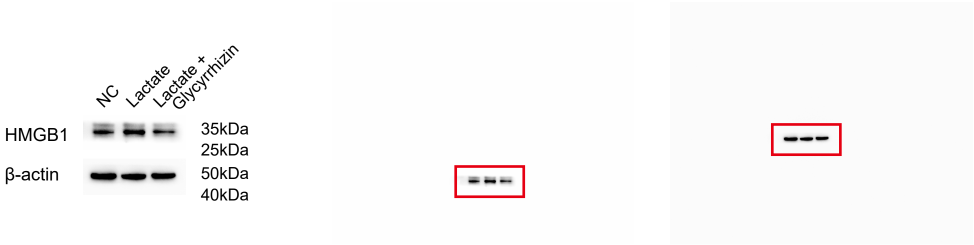

Figure 6E (RSC96)

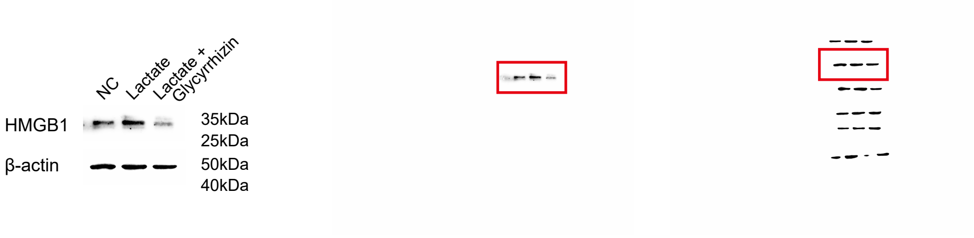

Supplement: Supplementary file 3 — Raw WB data [file 41419_2025_7543_MOESM3_ESM.pdf]
